# Trends in malignant intraductal papillary mucinous neoplasm in US adults from 1990 to 2010: a SEER database analysis

**DOI:** 10.1093/gastro/gov066

**Published:** 2016-01-27

**Authors:** Thomas R. McCarty, Basile Njei

**Affiliations:** ^1^Department of Internal Medicine, Yale University School of Medicine, New Haven, CT, USA,; ^2^Section of Digestive Diseases, Yale University School of Medicine, New Haven, CT, USA and; ^3^Investigative Medicine Program, Yale Center of Clinical Investigation, New Haven, CT, USA

**Keywords:** intraductal papillary mucinous neoplasm (IPMN), malignant transformation, incidence, long-term survival

## Abstract

**Background:** Intraductal papillary mucinous neoplasms (IPMNs) are precancerous lesions with a well-described adenoma-carcinoma sequence. Although the risk of malignant transformation has been well studied, data on trends in long-term survival and important prognostic factors associated with survival in malignant IPMN are lacking.

**Methods:** The Surveillance, Epidemiology, and End Results (SEER) database was queried to identify patients with confirmed malignant IPMN based upon pathologic diagnosis or radiographic evidence concerning for malignant potential. Median survival and age-adjusted incidence were calculated. Cox proportional hazard regression was used to determine independent mortality factors.

**Results:** Based upon the SEER database query, 2651 patients were diagnosed with malignant IPMN between 1990 and 2010. The age-adjusted incidence of IPMN in 1990 was 0.361 per 100 000 persons (95% confidence interval [CI]: 0.285–0.451) with a steady decline observed through 2010 (0.135 per 100 000 persons, 95% CI: 0.098–0.186). A total of 564 patients (21.3%) underwent a surgical procedure, though the number of patients who underwent surgery from 1990 to 2010 also decreased (1990–1995, *n* = 132 to 2006–2010, *n* = 96, respectively). The overall median survival was 4 months and remained relatively stable from 1990 to 2010. Performance of surgery (HR: 0.45, 95% CI: 0.40–0.53, *P* < 0.001) was associated with a decreased risk of death.

**Conclusion:** A significant decrease in the incidence of malignant IPMN was seen from 1990 to 2010. There was also no improvement observed in long-term survival. The small percentage of eligible cases receiving surgical treatment suggests that there is room for further improvement in survival, with increased utilization of surgery.

## Introduction

Intraductal papillary mucinous neoplasm (IPMN) of the pancreas is a type of neoplasm within the pancreatic ducts that is characterized by the production of thick, mucinous fluid [1–9]. These intraductal neoplasms may progress to invasive cancer—transforming from a benign to a malignant tumor if left untreated [10,11]. The tumor was first described in 1982 by Ohashi *et al.* in four patients with pancreatic carcinoma [12]. These patients were noted to have dilated main pancreatic ducts, patulous ampullary orifices, and mucus secretion from the pancreatic duct leading to the diagnosis. In 1996, the World Health Organization (WHO) introduced the term “intraductal papillary mucinous tumor”, which was then renamed IPMN in 2000 [2,13]. Since that time, the diagnosis and management of IPMN has received extensive recognition and scrutiny from the scientific and medical community, resulting in varied diagnostic and management algorithms. With this robust interest has come a dramatic increase in the number of IPMN diagnoses and surgical resections [11].

Although the true prevalence of IPMN is unknown at present, it has been estimated to range from 0.8 per 100 000 persons to as high as 10% of the population aged 70 years or older [14]. Other studies have suggested pancreatic cystic pathology to be present in up to 25% of patients at the time of autopsy, although it is important to note 97% of these lesions were benign [15]. The incidence of these mucin-producing epithelial cell tumors of the exocrine pancreas has been reported to be on the rise [16]. More recently, a study of reported IPMN incidence using data collected from Olmsted County, Minnesota, between 1985 and 2005, showed a 14-fold increase in the age- and sex-adjusted incidence of IPMN (0.008 to 0.32 per 100 000 persons, respectively) [17].

While this data may be extrapolated to larger cohorts to reflect the US population, no study has yet assessed whether the incidence of IPMN has truly increased on a national or large-scale level. Additionally, patient demographic data, as well as IPMN characteristics, are lacking. Most importantly, the disease-related morbidity and mortality of IPMN remains unclear, as does the role of surgical intervention. Current consensus guidelines recommend surgical resection for all main duct IPMNs, as determined radiographically by either main pancreatic duct dilatation ≥10 mm on computed tomography (CT) or magnetic resonance imaging (MRI) or borderline ductal dilatation of 5–9 mm with subsequent endoscopic ultrasound (EUS) confirmation of main duct involvement (i.e. thickened walls or intraductal mucin or nodules) [18,19]. Despite international consensus guidelines on the management of IPMN and mucinous cystic neoplasm of the pancreas, current guiding principles use vague descriptions and categories with no recommendations for treating IPMN in multi-morbid patients representative of everyday clinical practice [18–20]. With this gap in understanding and inability to transfer existing tumor knowledge to clinical practice, the true impact of IPMN remains unknown.

The primary aim of this study was to analyze and evaluate long-term survival trends of malignant IPMN in US adults and identify independent predictors of disease-related mortality. A secondary aim was to examine comparatively the survival patterns of IPMN in patients who underwent surgical intervention versus those patients who did not undergo surgery.

## Subjects and methods

### Data source

A retrospective cohort study was performed using data from the SEER database (available at www.seer.cancer.gov), based on the November 2011 submission. The SEER database is derived from 18 cancer registries representing approximately 28% of the US population and is maintained by the National Cancer Institute. The SEER dataset includes information on patient demographics, tumor and disease characteristics, cancer-associated treatments, use of cancer-directed surgery, and survival for individuals with cancer. Surgical interventions are coded in the SEER database as a separate variable and indicate if an operation was performed and if it was recommended or not. A surgical procedure directed at the primary site is also coded as a separate variable.

### Study population

The SEER database was queried for all cases of IPMN using tumor site codes with corresponding ICD-9 codes diagnosed between 1990 and 2010. Histologic codes for malignant neoplasm involving head of the pancreas, body of pancreas, tail of pancreas, other specified sites, and unspecified sites (157.0-157.9) were included in the search. Patients with another malignant primary tumor, diagnosed within five years prior to IPMN diagnosis, were excluded to minimize the chance that metastatic disease to the pancreas had been misdiagnosed as IPMN. To ensure uniform cancer staging classification across all study years, we used the SEER historic stage, which provides consistent definitions over time, as opposed to American Joint Committee on Cancer staging that is more commonly used in clinical settings but is not easily available for many of the years analyzed. The SEER historic stages were: localized (confined to primary site), regional (spread to regional lymph nodes), and distant (cancer had metastasized). Tumor location was defined as head of the pancreas, body, tail, multiple sites, pancreatic duct, and other. Patients diagnosed within one month prior to death (including patients diagnosed at autopsy or by death certificate) were excluded.

### Statistical analyses

We obtained SEER frequency, incidence, and survival data using SEER*Stat software, version 8.12. All rates were age adjusted to the 2000 US standard population. The National Cancer Institute’s Joinpoint Regression Analysis program (version 3.5) was used to examine trends in IPMN incidence and mortality. Incidence and mortality data were modeled in segmented log-linear form. The study population was divided into quartiles based upon year of diagnosis: 1990–1995, 1996–2000, 2001–2005, and 2006–2010. Mean and median values were used to describe continuous data, with discrete variables displayed as totals and frequencies. Median survival and survival rates were calculated overall and for each quartile. Trends in ordinal data were evaluated using the linear-by-linear association test. The linear-by-linear test of trend offers a measure of significance for ordinal variables (quartiles ordered from lowest to highest). Unless otherwise specified, the *P*-values reported for trend analysis refer to comparison among all of the four quartiles. A linear data analysis on annual incidence was also calculated and performed to analyze the trends in different age groups by sex and by race.

Subgroup analyses by treatment modality and stage at diagnosis were performed. Cumulative survival rates were calculated using the method of Kaplan-Meier, and survival curves were compared using the log-rank test. Univariate and multivariate Cox proportional hazard regression was used to determine independent predictors of mortality. Covariates with a *P*-value <0.1 on univariate analysis were entered into multivariate analysis. Covariates analyzed included age, sex, ethnicity (white, black, American Indian/Alaskan native and Asian or Pacific islander), tumor histology, tumor grade, stage of disease, and treatment (surgery, surgery plus adjuvant radiotherapy, or neither). All reported *P*-values were 2-tailed, and *P* < 0.05 was considered statistically significant for all tests. All analyses were performed using SEER*Stat and IBM SPSS version 20.0.

## Results

### Patient and intraductal papillary mucinous neoplasm characteristics

Using the SEER database, 2651 patients diagnosed with malignant IPMN between 1990 and 2010 met our inclusion criteria. Demographic and pathologic characteristics of the overall study population are shown in Table 1. The mean age of patients at the time of diagnosis was 67.9 years. Most patients were white (*n* = 2183; 82.3%). The proportion of female to male was 1.06. A majority of patients were >50 years of age at the time of diagnosis (<50 years, 7.2%; 50–69 years, 43.6% and ≥70 years, 49.2%, respectively). Overall, an overwhelming majority of patients had distant disease (*n* = 1626; 61.7%) as classified by the SEER historic stage. The number of patients diagnosed with malignant IPMN remained relatively constant over the study period, although the largest number were diagnosed during 2001–2005. Head of the pancreas and other (sites not otherwise specified from the categories as defined) were the two most common sites of tumor location (head [*n* = 957; 36.1%] and other [*n* = 963; 36.3%]).

**Table 1:** Characteristics for patients with intraductal papillary mucinous neoplasm (*n* = 2651)

Characteristics	No. of patients	Percentage (%)
Age at diagnosis (years)		
<50	192	7.2
50–69	1155	43.6
≥70	1304	49.2
Sex		
Female	1367	51.6
Male	1284	48.4
Race		
White	2183	82.3
Black	311	11.7
Others	157	6.0
SEER historic stage		
Localized	229	8.6
Regional	560	21.1
Distant	1636	61.7
Unstaged	226	8.5
Year of diagnosis		
1990–1995	607	22.9
1996–2000	580	21.9
2001–2005	933	35.2
2006–2010	531	20.0
Tumor location		
Head of pancreas	957	36.1
Body of pancreas	228	8.6
Tail of pancreas	327	12.3
Multiple sites/pancreatic duct	176	6.6
Other	963	36.3

### Intraductal papillary mucinous neoplasm incidence

From the beginning of the study period in 1990, the age-adjusted incidence of IPMN was 0.361 per 100 000 persons (95% CI: 0.285–0.451). This year marked the highest incidence of malignant IPMN cases, with a steady decline being observed through 2010 (0.135 per 100 000 persons, 95% CI: 0.098–0.186). Extrapolating this data further, the annual percent change from 1990 to 2010 demonstrated an age-adjusted incidence rate of −2.677, illustrating a declining incidence of malignant IPMN. This was calculated using one year for each endpoint. The overall percent change in age-adjusted incidence rates for 1990–2010, as specified above, resulted in an age-adjusted incidence of −45.653. Further data analyses are illustrated in Table 2. The annual incidence of malignant IPMN cases among male and female patients was also determined. The highest incident year was 2008 for males (0.105, 95% CI: 0.057–0.178) and 1992 for females (0.557, 95% CI: 0.068–0.432]) (Supplemental Tables 1 and 2). Further analyses of annual incidence by race showed the highest incidence of IPMN in 1991, 2010, and 1992 for white, black, and other races, respectively (0.311, 95% CI: 0.235–0.403; 0.581, 95% CI: 0.309–0.974; and 0.453, 95% CI: 0.190–0.899) (Supplemental Tables 3, 4, and 5).

**Table 2:** Age-adjusted incidences for 1990–2010

Year	Incidence	95% confidence interval	Standard error
1990–2010 % change	−45.653		
1990–2010 annual % change	−2.677*		
1990	0.361	0.285–0.451	0.041
1991	0.308	0.239–0.391	0.037
1992	0.37	0.294–0.459	0.041
1993	0.294	0.227–0.374	0.036
1994	0.29	0.224–0.368	0.036
1995	0.257	0.196–0.331	0.033
1996	0.292	0.227–0.370	0.035
1997	0.294	0.23–0.371	0.035
1998	0.261	0.201–0.333	0.033
1999	0.234	0.178–0.303	0.031
2000	0.28	0.219–0.354	0.033
2001	0.211	0.158–0.275	0.029
2002	0.298	0.235–0.373	0.034
2003	0.207	0.155–0.27	0.028
2004	0.16	0.116–0.215	0.024
2005	0.161	0.116–0.217	0.025
2006	0.197	0.148–0.258	0.027
2007	0.115	0.078–0.162	0.02
2008	0.143	0.102–0.194	0.023
2009	0.101	0.067–0.145	0.019
2010	0.137	0.098–0.186	0.022

Incidences are per 100 000 and age-adjusted to the 2000 US standard population.

% changes were calculated using one year for each endpoint.

Annual % changes were calculated using weighted least squares method.

*The annual % change is significantly different from zero (*P* < 0.05).

### Trends in intraductal papillary mucinous neoplasm-directed treatment

**Figure 1:**
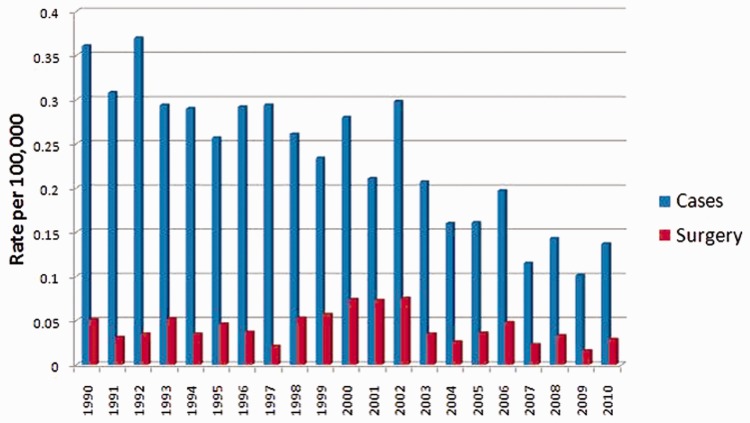
Rates of IPMN diagnosis and surgery from 1990 to 2010.

Of the 2651 patients included, 564 (21.3%) underwent a surgical procedure (Table 3). The mean age of patients undergoing surgery was 66.8 years with a slight female predominance (*n* = 292, 51.8%). The majority of surgical candidates were white as well (*n* = 453, 80.3%). SEER historic stage in this subset of surgical patients revealed regional staging in 50.5% with spread to regional lymph nodes. Head of the pancreas was the most common location of tumor in patients who underwent surgery (*n* = 202, 35.8%). The most common procedure type amongst this cohort included pancreatoduodenectomy or Whipple (*n* = 402, 71.3%). The number of patients who underwent surgery from 1990 to 2010 also decreased slightly when comparing an initial 6-year period to the final 5-year period of this study (*n* = 132 during 1990–1995 to *n* = 96 during 2006–2010). Rates of diagnosis and surgery are shown in Figure 1. A linear data analysis regarding surgical percentage of IPMN annually (not by quartiles) demonstrated that the highest percentages of surgery-related cases occurred in 1999 (30.12%) as shown in Supplemental Table 6.

**Table 3:** Characteristics of patients who underwent surgery: results of univariate analysis of surgery included (*n* = 564)

Characteristics	No. of patients	Percentage (%)	*P*-value
Age at diagnosis (years)			0.06
<50	42	7.4	
50–69	269	47.7	
≥70	253	44.9	
Sex			0.91
Female	292	51.8	
Male	272	48.2	
Race			0.02
White	453	80.3	
Black	63	11.2	
Others	48	8.5	
SEER historic stage			<0.001
Localized	131	23.2	
Regional	285	50.5	
Distant	136	24.1	
Unstaged	12	4.2	
Year of diagnosis			0.16
1990–1995	132	23.4	
1996–2000	137	24.3	
2001–2005	199	35.3	
2006–2010	96	17.0	
Tumor location			<0.001
Head of the pancreas	202	35.8	
Body of the pancreas	18	3.2	
Tail of the pancreas	39	6.9	
Multiple sites/pancreatic duct	23	4.1	
Other	282	50.0	
Procedure type			N/A
Partial pancreatectomy	94	16.7	
Total pancreatectomy	42	7.5	
Whipple	402	71.3	
Pancreatectomy, not otherwise specified	26	4.5	

N/A = not applicable.

On multivariable analysis, factors significantly associated with surgery included localized (adjusted odds ratio [OR]: 31.2, 95% CI: 16.1–52.3, *P* < 0.001) and regional disease (adjusted OR: 23.2, 95% CI: 15.9–50.5, *P* < 0.001). Additional factors included tumor location in the body of the pancreas (adjusted OR: 0.465, 95% CI: 0.267–0.809, *P* = 0.007) and year of diagnosis from 1996–2000 (adjusted OR: 1.447, 95% CI: 1.041–2.009, *P* = 0.028). Age at time of diagnosis, sex, and race were not associated with surgery (Table 4).

**Table 4:** Multivariable analysis of factors associated with surgery

Factor	Adjusted odds ratio	95% confidence interval	*P*-value
Age at diagnosis (years)			
≥70 *vs* <50	1.019	0.652–1.593	0.933
50–69 *vs* <50	0.668	0.426–1.048	0.079
Sex			
Male *vs* female	0.916	0.733–1.145	0.440
Race			
Black *vs* white	1.461	0.848–2.518	0.172
Others *vs* white	2.189	0.255–18.799	0.475
SEER historic stage			
Localized *vs* distant	31.2	16.1–52.3	<0.001
Regional *vs* distant	23.2	15.9–50.5	<0.001
Unstaged *vs* distant	1.3	0.55–2.29	0.72
Year of diagnosis			
1996–2000 *vs* 1990–1995	1.447	1.041–2.009	0.028
2001–2005 *vs* 1990–1995	1.205	0.893–1.627	0.223
2006–2010 *vs* 1990–1995	0.931	0.656–1.319	0.686
Tumor location			
Body *vs* head	0.465	0.267–0.809	0.007
Tail *vs* head	1.260	0.825–1.923	0.285
Multiple sites/pancreatic duct *vs* head	0.966	0.569–1.642	0.900
Other *vs* head	2.303	1.780–2.980	<0.001

### Survival trends

Cox proportional hazard regression was used to determine independent mortality factors (Table 5). Age at time of diagnosis ≥70 years was a significant prognostic factor of independent mortality (hazard ratio [HR]: 1.347, 95% CI: 1.156–1.570, *P* < 0.001). Distant disease based upon the SEER historic stage classification (HR: 1.348, 95% CI: 1.067–10.507, *P* = 0.038), year of diagnosis during 1996–2000 (HR: 1.150, 95% CI: 1.150–1.025, *P* = 0.018), and tumor location in the body of the pancreas (HR: 1.191, 95% CI: 1.046–1.355, *P* = 0.008) were also significant factors.

**Table 5:** Cox proportional hazards analyses of prognostic factors

Factor	Hazard ratio	95% confidence interval	*P*-value
Age at diagnosis (years)			
50–69 *vs* < 50	0.995	0.853–1.160	0.945
≥ 70 *vs* < 50	1.347	1.156–1.570	<0.001
Sex			
Male *vs* female	1.029	0.953–1.111	0.465
SEER historic stage			
Distant *vs* localized	3.348	1.067–10.507	0.038
Regional *vs* localized	1.391	0.443–4.371	0.572
Unstaged *vs* localized	1.789	0.571–5.599	0.318
Year of diagnosis			
1996–2000 *vs* 1990–1995	1.150	1.025–1.292	0.018
2001–2005 *vs* 1990–1995	1.020	0.918–1.133	0.714
2006–2010 *vs* 1990–1995	1.085	0.962–1.224	0.183
Tumor location			
Body *vs* head	1.020	0.881–1.181	0.788
Tail *vs* head	1.191	1.046–1.355	0.008
Multiple sites/pancreatic duct *vs* head	1.045	0.888–1.229	0.598
Other *vs* head	1.004	0.913–1.105	0.929
Surgery performed			
Yes *vs* no	0.453	0.401–0.512	<0.001

**Figure 2:**
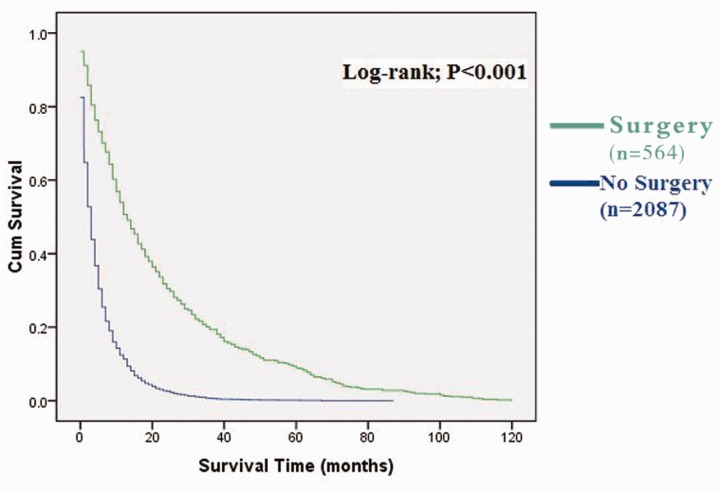
Kaplan-Meier analysis: cumulative survival rate of IPMN patients with and without surgery.

The overall median survival was 4 months (95% CI: 3.718–4.282). Despite a decreased incidence in malignant IPMN, the median survival remained constant throughout the reviewed time frame (1990–1995: 4 months, 95% CI: 3.389–4.611; 2006–2010: 4 months, 95% CI: 3.402–4.598). Patients who underwent surgery demonstrated a median survival time of 14 months (95% CI: 11.977–16.023). Non-surgical patients had a significantly lower mean survival time of 3 months (95% CI: 2.796–3.204, *P* < 0.001). Cumulative survival rates were calculated using the method of Kaplan-Meier and survival curve shown in Figure 2.

## Discussion

Previous local population-based studies have suggested an increase in the age and sex-adjusted incidence of all IPMN types. With more focus and IPMN-specific attention from those in the scientific community, we postulate improved management for benign IPMN. It is not surprising then that improved surveillance and watchful waiting strategies may have resulted in our study demonstrating a continual decline in the age-adjusted incidence of malignant IPMN from 1990 to 2010 [17]. The number of patients who underwent surgery from 1990 to 2010 also decreased as well. Despite this decline in the incidence of malignant IPMN, the median survival from 1990 to 2010 remained constant.

According to our data, older age at the time of diagnosis (>70 years), presence of distant disease, and location of tumor within the body of the pancreas had the highest associated mortality risk. Not surprisingly, median survival for patients who underwent surgical resection as compared with non-surgical IPMN demonstrated a significant survival advantage (14 months compared with 3 months, respectively). Despite the proven benefit of surgery (as demonstrated by our data), the number of patients with malignant IPMN cases undergoing surgery from 2006 to 2010 declined. A decline in surgical intervention may reflect worsened comorbidities in malignant IPMN individuals, resulting in fewer surgical candidates or other confounders not measured by this study. More likely, however, the observed decline in the number of malignant IPMN cases as well as less surgical intervention may be secondary to improved management of benign or borderline IPMN through increased surveillance programs or watchful waiting strategies.

With the shift in awareness to pancreatic cystic lesions, there has been a mounting challenge for clinicians to determine which of these lesions warrants surgical intervention, cytological sampling, ongoing surveillance, or simply reassurance [17]. Yet, prior to this study, there has not been a previous major attempt to characterize IPMN beyond diagnostic imaging findings on a national scale. More accurate diagnosis and prediction of malignancy represent major challenges in current IPMN management as well as management of pancreatic cysts in general [21]. In translation to clinical practice, we hope this study will aid clinicians by identifying further independent mortality factors associated with IPMN in addition to the known imaging findings upon which the guidelines currently rely [18,19,21]. Ultimately, while current guidelines are adept at identifying which patients may benefit from surgical intervention through imaging modalities, predicting malignant transformation of an IPMN lesion will require incorporation of clinical, imaging, and multiple EUS/FNA features [21,22]. Radiographic evidence has certainly improved identification of benign or borderline IPMN, thus reducing the total number of malignant cases between 1990 and 2010. With current guidelines in place based upon IPMN size or main duct branch involvement, adoption of a more aggressive surgical approach encompassing age, presence of distant disease, and tumor within the body of the pancreas may demonstrate further improvement in long-term survival.

Strengths of this study include the large number of patients reviewed and the availability of sufficient trend data for survival estimates as well as predictors of survival. Interpretation of our results does possess inherent limitations because the SEER database lacks information on method of diagnosis (i.e. type of imaging modality), patient comorbidities, and case-to-case specific surgery procedural data. It is also important to note that the SEER database does not capture benign tumors; therefore, these results cannot be applied to all cystic lesions and benign IPMNs [23].

Additionally, the distinction between IPMN and mucinous cystic neoplasm had not been clarified before 1999, so many lesions that were previously classified as mucinous cystic neoplasms may have been excluded. Given the nature of data used, it is also important to consider the possibility of patient selection bias, miscoding and missing, insufficient or inaccurate data entry. In response to this, studies have been performed showing that data were entered accurately in the SEER database and that misclassification bias is minimal [24–28]. However, if this was indeed thought to be the case, our data would suggest an even larger decline in the number of cases of IPMN. It has also been suggested that a decline in malignant IPMN may suggest improved screening prior to malignant conversion—with benign IPMN not being captured by the SEER registry [23].

In conclusion, the incidence of all types of IPMN has certainly increased as evidenced by daily clinical practice. Our results, however, have demonstrated a declining incidence of malignant IPMN in US adults, which suggest annual improvement of benign or borderline IPMN management by surgery or active surveillance. Additionally, no improvement was observed in overall long-term survival of malignant IPMN. The small percentage of eligible cases receiving surgical treatment may suggest an opportunity for further improvement in survival by increased utilization of surgery. Most importantly, the identification of high-risk patient characteristics alongside current imaging guidelines may serve to enhance the role of surgical intervention for a selected malignant IPMN population and translate into a more significant survival benefit.

*Conflict of interest statement*: none declared.

## Supplementary Material

Supplementary Data
