# Supplementary material for: Trends in malignant intraductal papillary mucinous neoplasm in US adults from 1990 to 2010: a SEER database analysis
Source: Gastroenterol Rep (Oxf). 2016 Jan 27;4(2):113–8. doi: 10.1093/gastro/gov066 (PMC4863191; doi:10.1093/gastro/gov066)
Supplement: Supplementary Data [file supp_4_2_113__index.html]

Trends in malignant intraductal papillary mucinous neoplasm in US adults from 1990 to 2010: a SEER database analysis — Trends in malignant intraductal papillary mucinous neoplasm in US adults from 1990 to 2010: a SEER database analysis — Supplementary Data 

# Trends in malignant intraductal papillary mucinous neoplasm in US adults from 1990 to 2010: a SEER database analysis

## Supplementary Data

files

- Supplementary Data - docx file
