# Supplementary material for: Trends in malignant intraductal papillary mucinous neoplasm in US adults from 1990 to 2010: a SEER database analysis
Source: Gastroenterol Rep (Oxf). 2016 Jan 27;4(2):113–8. doi: 10.1093/gastro/gov066 (PMC4863191; doi:10.1093/gastro/gov066)
Supplement: Supplementary Data [file supp_gov066_Supplemental_Tables.docx]

**Supplemental Table 1:** Annual incidence among males - Overall annual incidence of malignant IPMN by male gender.

| **Year** | **Incidence (per 100,000)** | **Lower 95% CI** | **Upper 95% CI** | **Standard error** |
| --- | --- | --- | --- | --- |
| 1990 | 0.019 | 0.002 | 0.07 | 0.013 |
| 1991 | 0.049 | 0.016 | 0.114 | 0.022 |
| 1992 | 0.03 | 0.006 | 0.086 | 0.017 |
| 1993 | 0.06 | 0.019 | 0.137 | 0.028 |
| 1994 | 0.018 | 0.002 | 0.066 | 0.013 |
| 1995 | 0.021 | 0.002 | 0.072 | 0.015 |
| 1996 | 0.036 | 0.01 | 0.092 | 0.018 |
| 1997 | 0.016 | 0.002 | 0.06 | 0.011 |
| 1998 | 0.025 | 0.005 | 0.074 | 0.015 |
| 1999 | 0.032 | 0.006 | 0.089 | 0.019 |
| 2000 | 0.015 | 0.002 | 0.056 | 0.011 |
| 2001 | 0.034 | 0.009 | 0.087 | 0.017 |
| 2002 | 0.054 | 0.02 | 0.117 | 0.022 |
| 2003 | 0.083 | 0.04 | 0.153 | 0.027 |
| 2004 | 0.051 | 0.018 | 0.11 | 0.021 |
| 2005 | 0.055 | 0.022 | 0.114 | 0.021 |
| 2006 | 0.027 | 0.006 | 0.077 | 0.016 |
| 2007 | 0.067 | 0.028 | 0.131 | 0.024 |
| 2008 | 0.105 | 0.057 | 0.178 | 0.029 |
| 2009 | 0.05 | 0.02 | 0.105 | 0.019 |
| 2010 | 0.048 | 0.018 | 0.1 | 0.019 |

**Supplemental Table 2:** Annual incidence among females - Overall annual incidence of malignant IPMN by female gender.

| **Year** | **Incidence (per 100,000)** | **Lower 95% CI** | **Upper 95% CI** | **Standard error** |
| --- | --- | --- | --- | --- |
| 1990 | 0.331 | 0.234 | 0.453 | 0.054 |
| 1991 | 0.495 | 0.378 | 0.638 | 0.064 |
| 1992 | 0.557 | 0.432 | 0.708 | 0.068 |
| 1993 | 0.389 | 0.287 | 0.515 | 0.056 |
| 1994 | 0.439 | 0.331 | 0.571 | 0.059 |
| 1995 | 0.43 | 0.324 | 0.559 | 0.058 |
| 1996 | 0.338 | 0.247 | 0.452 | 0.05 |
| 1997 | 0.411 | 0.309 | 0.535 | 0.056 |
| 1998 | 0.296 | 0.212 | 0.403 | 0.046 |
| 1999 | 0.392 | 0.294 | 0.512 | 0.054 |
| 2000 | 0.399 | 0.301 | 0.519 | 0.054 |
| 2001 | 0.326 | 0.238 | 0.435 | 0.048 |
| 2002 | 0.284 | 0.204 | 0.386 | 0.045 |
| 2003 | 0.286 | 0.206 | 0.388 | 0.044 |
| 2004 | 0.348 | 0.258 | 0.46 | 0.049 |
| 2005 | 0.262 | 0.186 | 0.359 | 0.042 |
| 2006 | 0.298 | 0.217 | 0.399 | 0.044 |
| 2007 | 0.344 | 0.257 | 0.451 | 0.047 |
| 2008 | 0.296 | 0.215 | 0.396 | 0.044 |
| 2009 | 0.414 | 0.32 | 0.528 | 0.051 |
| 2010 | 0.508 | 0.403 | 0.633 | 0.057 |

**Supplemental Table 3:** Annual incidence among white race - Overall annual incidence of malignant IPMN by white race.

| **Year** | **Incidence (per 100,000)** | **Lower 95% CI** | **Upper 95% CI** | **Standard error** |
| --- | --- | --- | --- | --- |
| 1990 | 0.183 | 0.126 | 0.258 | 0.032 |
| 1991 | 0.311 | 0.235 | 0.403 | 0.041 |
| 1992 | 0.304 | 0.229 | 0.396 | 0.041 |
| 1993 | 0.25 | 0.183 | 0.332 | 0.036 |
| 1994 | 0.222 | 0.161 | 0.299 | 0.034 |
| 1995 | 0.244 | 0.179 | 0.323 | 0.035 |
| 1996 | 0.184 | 0.13 | 0.254 | 0.03 |
| 1997 | 0.224 | 0.163 | 0.299 | 0.033 |
| 1998 | 0.172 | 0.12 | 0.239 | 0.029 |
| 1999 | 0.219 | 0.16 | 0.293 | 0.033 |
| 2000 | 0.211 | 0.153 | 0.283 | 0.032 |
| 2001 | 0.191 | 0.136 | 0.261 | 0.03 |
| 2002 | 0.166 | 0.115 | 0.231 | 0.028 |
| 2003 | 0.196 | 0.141 | 0.265 | 0.03 |
| 2004 | 0.21 | 0.153 | 0.283 | 0.032 |
| 2005 | 0.167 | 0.117 | 0.231 | 0.028 |
| 2006 | 0.163 | 0.114 | 0.227 | 0.027 |
| 2007 | 0.168 | 0.118 | 0.232 | 0.028 |
| 2008 | 0.193 | 0.139 | 0.26 | 0.029 |
| 2009 | 0.212 | 0.156 | 0.281 | 0.03 |
| 2010 | 0.276 | 0.212 | 0.353 | 0.035 |

**Supplemental Table 4:** Annual incidence among black race- Overall annual incidence of malignant IPMN by black race.

| **Year** | **Incidence (per 100,000)** | **Lower 95% CI** | **Upper 95% CI** | **Standard error** |
| --- | --- | --- | --- | --- |
| 1990 | 0.259 | 0.081 | 0.61 | 0.119 |
| 1991 | 0.321 | 0.097 | 0.739 | 0.151 |
| 1992 | 0.444 | 0.183 | 0.879 | 0.164 |
| 1993 | 0.168 | 0.034 | 0.475 | 0.099 |
| 1994 | 0.364 | 0.143 | 0.746 | 0.141 |
| 1995 | 0.461 | 0.197 | 0.89 | 0.165 |
| 1996 | 0.49 | 0.219 | 0.919 | 0.167 |
| 1997 | 0.243 | 0.077 | 0.559 | 0.111 |
| 1998 | 0.338 | 0.122 | 0.712 | 0.14 |
| 1999 | 0.446 | 0.199 | 0.838 | 0.152 |
| 2000 | 0.386 | 0.168 | 0.742 | 0.135 |
| 2001 | 0.32 | 0.123 | 0.658 | 0.126 |
| 2002 | 0.442 | 0.208 | 0.808 | 0.143 |
| 2003 | 0.421 | 0.177 | 0.814 | 0.153 |
| 2004 | 0.394 | 0.172 | 0.749 | 0.137 |
| 2005 | 0.207 | 0.065 | 0.477 | 0.095 |
| 2006 | 0.326 | 0.145 | 0.624 | 0.112 |
| 2007 | 0.552 | 0.287 | 0.943 | 0.158 |
| 2008 | 0.326 | 0.153 | 0.606 | 0.105 |
| 2009 | 0.515 | 0.278 | 0.863 | 0.14 |
| 2010 | 0.581 | 0.309 | 0.974 | 0.161 |

**Supplemental Table 5:** Annual incidence among other races - Overall annual incidence of malignant IPMN by other races (American Indian/Alaskan Native and Asian or Pacific Islander).

| **Year** | **Incidence (per 100,000)** | **Lower 95% CI** | **Upper 95% CI** | **Standard error** |
| --- | --- | --- | --- | --- |
| 1990 | 0.198 | 0.041 | 0.562 | 0.114 |
| 1991 | 0.222 | 0.06 | 0.571 | 0.112 |
| 1992 | 0.453 | 0.19 | 0.899 | 0.165 |
| 1993 | 0.197 | 0.053 | 0.509 | 0.099 |
| 1994 | 0.388 | 0.167 | 0.764 | 0.138 |
| 1995 | 0.114 | 0.014 | 0.387 | 0.081 |
| 1996 | 0.2 | 0.053 | 0.505 | 0.102 |
| 1997 | 0.324 | 0.129 | 0.663 | 0.124 |
| 1998 | 0.088 | 0.011 | 0.31 | 0.062 |
| 1999 | 0.084 | 0.01 | 0.297 | 0.059 |
| 2000 | 0.209 | 0.067 | 0.482 | 0.094 |
| 2001 | 0.116 | 0.024 | 0.333 | 0.067 |
| 2002 | 0.065 | 0.008 | 0.241 | 0.046 |
| 2003 | 0.066 | 0.008 | 0.24 | 0.047 |
| 2004 | 0.102 | 0.021 | 0.295 | 0.059 |
| 2005 | 0.146 | 0.039 | 0.364 | 0.073 |
| 2006 | 0.13 | 0.035 | 0.329 | 0.065 |
| 2007 | 0.336 | 0.166 | 0.603 | 0.103 |
| 2008 | 0.174 | 0.063 | 0.38 | 0.072 |
| 2009 | 0.281 | 0.133 | 0.52 | 0.09 |
| 2010 | 0.277 | 0.131 | 0.513 | 0.09 |

**Supplemental Table 6:** Annual surgical percentage of IPMN cases per total number of cases

| Year | Percentage (%) |
| --- | --- |
| 1990 | 22.11 |
| 1991 | 26.92 |
| 1992 | 20.86 |
| 1993 | 22.22 |
| 1994 | 13.86 |
| 1995 | 26.74 |
| 1996 | 15.46 |
| 1997 | 15.63 |
| 1998 | 29.13 |
| 1999 | 30.12 |
| 2000 | 25.87 |
| 2001 | 29.51 |
| 2002 | 25.21 |
| 2003 | 12.5 |
| 2004 | 14.97 |
| 2005 | 23.03 |
| 2006 | 22.6 |
| 2007 | 20.77 |
| 2008 | 18.35 |
| 2009 | 16.67 |
| 2010 | 21.27 |
